# Facebook Addiction Disorder (FAD) among German students—A longitudinal approach

**DOI:** 10.1371/journal.pone.0189719

**Published:** 2017-12-14

**Authors:** Julia Brailovskaia, Jürgen Margraf

**Affiliations:** Mental Health Research and Treatment Center, Ruhr-Universität Bochum, Bochum, Germany; Swansea University, UNITED KINGDOM

## Abstract

The present study aimed to investigate Facebook Addiction Disorder (FAD) in a German student sample over a period of one year. While mean FAD level did not increase during the investigation period, a significant increase was shown in the number of participants reaching the critical cutoff score. FAD was significantly positively related to the personality trait narcissism and to negative mental health variables (depression, anxiety, and stress symptoms). Furthermore, FAD fully mediated the significant positive relationship between narcissism and stress symptoms, which demonstrates that narcissistic people can be specifically at risk to develop FAD. Present results give a first overview of FAD in Germany. Practical applications for future studies and limitations of present results are discussed.

## Introduction

Excessive consumption of psychoactive chemicals, like alcohol and other drugs, is well known to trigger addictive behavior. However, behavioral (i.e., non-substance) addictions are still a contentious topic. So far, only pathological gambling has been recognized as a formal psychiatric disorder in the Diagnostic and Statistical Manual of Mental Disorders (5^th^ ed., DSM-5; [1]). Additionally, Internet gaming disorder was included in the “Emerging Measures and Models” section of the DSM-5 [1, 2]. Thus, there is a high need for further rigorous research and for studies finding significant evidence in the area of behavioral addictions [3, 4]. Considering the great importance of social media in the daily life of people today, a number of recent studies have focused on further problematic media use (e.g., [5, 6]). While some studies investigated general Internet addiction [7–9] and reported, for example, a positive association between problematic Internet use, depression and anxiety symptoms, other studies have addressed addiction to social networking sites (SNSs) [10], especially to the popular international SNS Facebook [4, 11, 12].

Currently, Facebook has over 2.1 billion members [13]. For many of them Facebook use has become a significant part of daily life [14], and some of them seem to lose control over their Facebook use and to develop a strong psychological need to stay online, despite the possible negative consequences of this behavior [15]–so-called Facebook Addiction Disorder (FAD) [16]. FAD is defined by six typical characteristics of addiction disorders: salience (e.g., permanent thinking of Facebook use), tolerance (e.g., requiring increasing time on Facebook to achieve previous positive using effect), mood modification (e.g., mood improvement by Facebook use), relapse (reverting to earlier use pattern after ineffective attempts to reduce Facebook use), withdrawal symptoms (e.g., becoming nervous without possibility to use Facebook), and conflict (e.g., interpersonal problems caused by intensive Facebook use) [11, 15, 17].

While FAD was found to be positively associated with male gender, circadian rhythm (late bedtimes and rising times on weekdays and weekend), insomnia, depression and anxiety symptoms, its relationship with age, openness, agreeableness, and conscientiousness was negative [11, 12, 15, 18–20]. Błachnio et al. [21] investigated FAD in different countries. They described the highest FAD levels in China and the lowest in Poland. Thus, available studies have demonstrated FAD to occur in different populations and to be associated with various factors, such as demographic variables, mental health variables, and personality traits. However, these results are not sufficient to officially recognize FAD as a behavioral addiction. One reason is the cross-sectional nature of the present studies, which provide little evidence about the development and maintenance of FAD. Therefore, longitudinal research is needed to gain further insight into the epidemiology of FAD and to understand which factors are associated with problematic Facebook use. This knowledge is necessary for the elaboration of intervention programs aimed to protect mental (see [22]).

Furthermore, many studies considering FAD came from countries such as Norway, Malaysia, and Turkey (e.g., [11, 19, 20, 23, 24]). In contrast, even though Facebook use has become an integral component of everyday life of a large part of the German population, especially younger people [25], only little attention has been paid to FAD in Germany.

**Fig 1:**
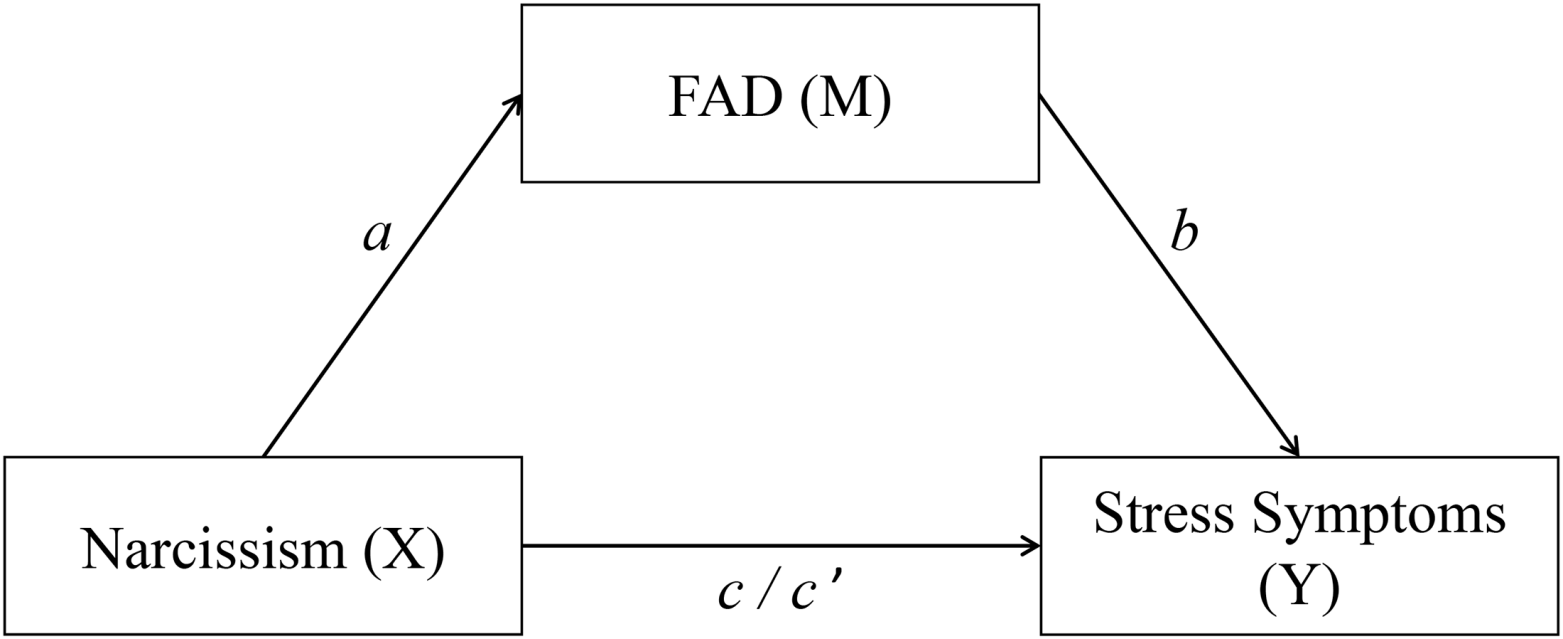
Mediation model with narcissism as predictor (X), FAD as mediator (M), and stress symptoms as outcome (Y) (Hypothesis 5). *c* = total effect, *c’* = direct effect.

Therefore, the main aim of the present study was to investigate the epidemiology of FAD over the course of one year (two measurement time points) in a German sample. Considering the lack of knowledge about the development of FAD, this investigation had primarily an exploratory character (see [4]). A second issue was to determinate the associations between FAD and different mental health variables, as well as physical health (see Hypothesis 1 to Hypothesis 5) and to investigate whether these associations change over time. This approach should contribute to a better understanding of FAD. Considering earlier results which found a positive association between FAD and Facebook use, on the one hand, and depression, anxiety, and stress symptoms, on the other hand [11, 26, 27], we hypothesized to find a positive association between FAD and negative mental health (i.e., depression, anxiety, and stress symptoms) (Hypothesis 1). Shakya and Christakis [28] and Kross et al. [29] described persistent Facebook use to be negatively associated with positive variables like life satisfaction and physical health. Therefore, we further assumed to find a negative relationship between FAD and positive mental health variables (i.e., life satisfaction, social support) (Hypothesis 2), as well as physical health (Hypothesis 3). Additionally, we included the personality trait narcissism that has been frequently reported to be positively associated with intensive social media use (e.g., [30–32]) in our investigation. Typically, narcissistic people use Facebook for self-presentation and social interaction to satisfy their need for attention and admiration [33, 34]. If such individuals fail to receive the desired amount of attention, they often experience stress symptoms [35]. Therefore, we expected the personality trait narcissism to be positively related to FAD (Hypothesis 4). Moreover, we assumed that FAD can mediate the relationship between narcissism and stress symptoms (Hypothesis 5) (see Fig 1).

## Materials and methods

### Procedure and participants

The present study belongs to the ongoing BOOM (Bochum Optimism and Mental Health) research program which investigates risk and protective factors of mental health [36–39]. Since 2011, an invitation e-mail including a link to the baseline online survey is sent to all students enrolled at the Ruhr-Universität Bochum, a large German state university. At the end of the baseline survey, which includes questionnaires on different aspects of mental health and personality, participants are asked whether they agree to be included in the BOOM participant pool and to be contacted for further investigations. The participation at the BOOM online survey is voluntary and can be compensated by course credits.

In December 2015, a collective e-mail with a participation invitation and the link for the online survey was sent to a randomly collected sample of 300 persons from the BOOM student participant pool (first measurement time point, T1). The only requirement for participation was a current Facebook membership. In December 2016, those who had completed the first survey (N = 185) received a further e-mail invitation to the second online survey (second measurement time point, T2) which included the same questions as the survey at T1. In whole, 179 students (77.1% women) from different faculties and semesters (1.-2.: 41.3%, 3.-4.: 23.5%, 5.-6.: 13.4%, 7. ≤: 21.8%) completed both surveys (age (years): M = 22.52, SD = 5.00, range: 17–58). While 46.3% of the participants were single, 49.2% of them lived in a steady relationship, and 4.5% of them were married. The Ethics Committee of the Ruhr-Universität Bochum approved the implementation of the present study. We followed all national regulations and laws regarding human subjects research, and obtained the required permission to conduct the present study. Participants were properly instructed and gave online informed consent to participate. A priori conducted power analyses (G*Power program, version 3.1) showed that the sample size was sufficient for valid results (power > .80, α = .05, effect size f^2^ = 0.15) (cf., [40]). The dataset used in the present study is available in S1 Dataset.

### Measures

#### Mental health

**Life satisfaction**. The unidimensional Satisfaction with Life Scale (SWLS) [41] measured global life satisfaction with five items (e.g., “In most ways, my life is close to my ideal.”) rated on a 7-point Likert scale (1 = strongly disagree, 7 = strongly agree). Higher scores indicate higher levels of life satisfaction. The total score can range from seven to 35. The SWLS has good psychometric properties. Its convergent and discriminant validity has been demonstrated earlier [36, 42]. The internal scale reliability has been found to be Cronbach’s α = .92 [43]. Current scale reliability was α_T1_ = .89/α_T2_ = .89.

**Social support**. To measure subjective perceived or anticipated social support the brief unidimensional version of the Questionnaire Social Support (F-SozU K-14) [44] was used. It consists of 14 items (e.g., “I experience a lot of understanding and security from others.”) rated on a 5-point Likert scale (1 = not true at all, 5 = very true). The higher the total score, the higher the level of perceived or anticipated social support. The total score can range from 14 to 70. This instrument has good values of convergent and discriminant validity, as well as good retest-reliability. The internal scale reliability has been reported to be α = .94 [36, 44]. Current internal reliability was α_T1_ = .91/α_T2_ = .93.

**Depression, anxiety, stress**. The Depression Anxiety Stress Scales 21 (DASS-21) [45], a brief version of the DASS-42, measured depression, anxiety, and stress symptoms over the previous week on three 7-item subscales (i.e., scale depression, “I couldn’t seem to experience any positive feeling at all.”; scale anxiety, “I felt scared without any good reason.”; scale stress, “I tended to over-react to situations.”) rated on a 4-point Likert scale (0 = did not apply to me at all, 3 = applied to me very much or most of the time). Higher scores on the three scales indicate higher levels of depression, anxiety, and stress symptoms. The total score of each scale can range from zero to 21. The DASS-21 is a well-established instrument in non-clinical and clinical samples with similar good psychometric properties as the long 42-item version [46]. Its internal scale reliability has been reported to vary between the three scales (depression: α = .83; anxiety: α = .78; stress: α = .87) [47]. Current internal reliability was α_T1_ = .86/α_T2_ = .88 for the depression scale, α_T1_ = .80/α_T2_ = .79 for the anxiety scale, and α_T1_ = .87/α_T2_ = .88 for the stress scale.

**Facebook Addiction Disorder (FAD)**. FAD adhering to a time frame of the last year was assessed by the brief version of the Bergen Facebook Addiction Scale (BFAS) [15] which includes six items (e.g., “Become restless or troubled if you have been prohibited from using Facebook?”) according to the six core addiction features (i.e., salience, tolerance, mood modification, relapse, withdrawal, conflict) rated on a 5-point Likert scale (1 = very rarely, 5 = very often). Higher scores reflect higher levels of FAD. The overall score can range from six to 30. The 6-item version of the BFAS has been demonstrated to have similar good psychometric properties as the long 18-item version. The internal scale reliability of the brief version has been found to be α = .83/.86 [15, 20, 48]. Current scale reliability was α_T1_ = .73/α_T2_ = .82. Until now, specific cutoff scores to categorize FAD have been rarely investigated. Considering research on other addictions, Andreassen et al. [15] suggested two possible categorization approaches for problematic BFAS values: a more liberal approach concerning a polythetic scoring scheme (cutoff score: ≥ 3 on at least four of the six items), or a more conservative approach concerning a monothetic scoring scheme (cutoff score: ≥ 3 on all six items).

#### Narcissism

To assess the personality trait narcissism, the brief Narcissistic Personality Inventory (NPI-13) [49] consisting of 13 forced-choice format items (0 = low narcissism, e.g., “I don’t like it when I find myself manipulating people.”, 1 = high narcissism, e.g., “I find it easy to manipulate people.”) was used. The higher the total score, the higher the level of narcissism. The total score can range from zero to 13. The NPI-13 has been demonstrated to have similarly good psychometric properties as the full-length 40-item version and to preserve its conceptual breath [50, 51]. It provides a total score as well as three subscale scores (i.e., leadership/authority (LA), grandiose exhibitionisms (GE), entitlement/exploitativeness (EE), see [52]). The present study focused only on the total narcissism score. Earlier studies reported an internal scale reliability of α = .67/.73 [49, 51]. Current internal reliability was α_T1_ = .53/α_T2_ = .60.

#### Physical health

The EuroQuol Visual Analogue Scale (EQ VAS) [53, 54]–a visual analogue scale ranging from 0 (worst imaginable health state) to 100 (best imaginable health state)–assessed participants’ overall current physical health status. Higher scores indicate higher levels of physical health. Validity of the EQ VAS has been demonstrated by earlier research [55].

#### Media use

Frequency of general Internet use and of SNSs use was rated on a 7-point Likert scale (0 = never, 6 = more than once a day). Higher scores present higher use frequency. Additionally, participants were asked whether they are also members of other SNSs than Facebook (i.e., Twitter, Instagram, Tumblr, or any other SNS: 0 = no, 1 = yes) and how many SNSs they use in whole [34].

### Statistical analyses

Statistical analyses were conducted with the Statistical Package for the Social Sciences (SPSS) 24 and the macro Process version 2.16.1 (www.processmacro.org/index.html). After descriptive analyses of the investigated variables, their possible changes between T1 and T2 were assessed by repeated measures analysis of variance (within-subjects ANOVA). Associations between the investigated variables were assessed by calculating zero-order bivariate correlations and multiple linear regression analyses. Next, the mediation model presented in Fig 1 was analyzed. The basic relationship between narcissism (predictor, X) and stress symptoms (outcome, Y) was denoted by *c* (the total effect). The path of narcissism to FAD (mediator, M) was denoted by *a*, and the path of FAD to stress was denoted by *b*. The indirect effect was represented by the combined effect of path *a* and path *b*, and path *c’* denoted the direct effect of narcissism to stress symptoms after the inclusion of FAD in the model. The mediation effect was assessed by the bootstrapping procedure (10.000 samples) which provides accelerated confidence intervals (CI 95%). Considering the shortcomings of the effect size kappa-squared (κ^2^) commonly used in mediation analyses, P_M_ (the ration of the indirect effect to the total effect) was used as the mediation effect measure [56].

## Results

### Descriptive analyses and comparison between T1 and T2

All investigated variables were close to normally distributed (indicated by Kolmogorov-Smirnov test, analyses of skew, kurtosis, and histogram). Tables 1 and 2 present their descriptive values. Moreover, Table 1 shows the results of the within-subject ANOVAs comparing T1 and T2 values. While values for physical health decreased significantly (partial eta^2^ = .04), the values of depression symptoms (partial eta^2^ = .06) and the mean number of used SNSs (partial eta^2^ = .02) increased significantly. The described effects were small.

**Table 1:** Descriptive statistics and mean comparison between T1 and T2 values of personality, physical and mental health, and media use variables (within-subjects ANOVA).

	T1		T2			
	M (SD)	Min–Max	M (SD)	Min–Max	F	p
NPI-13	3.64 (2.19)	0–10	3.77 (2.37)	0–11	1.289	.258
EQ VAS	79.98 (15.34)	20–100	76.44 (18.87)	0–100	6.928	.009
**Mental health**						
SWLS	26.00 (5.84)	8–35	26.30 (5.78)	8–35	.686	.409
F-SozU	61.80 (8.00)	33–70	61.78 (8.58)	33–70	.003	.955
DASS: Depression	3.56 (3.73)	0–20	4.53 (4.31)	0–21	11.139	.001
DASS: Anxiety	2.79 (3.15)	0–16	2.82 (3.28)	0–16	.016	.899
DASS: Stress	5.70 (4.09)	0–19	6.28 (4.59)	0–21	2.682	.103
BFAS	9.53 (3.18)	6–23	9.77 (3.86)	6–28	.767	.382
**Media use**						
Internet use	5.65 (.74)	2–6	5.70 (.71)	1–6	.599	.440
SNSs use	4.71 (1.04)	1–6	4.60 (1.37)	1–6	1.702	.194
Used SNSs	1.49 (.75)	1–5	1.59 (.76)	1–4	4.343	.039

N = 179; M = Mean; SD = Standard Deviation; Min = Minimum; Max = Maximum; NPI = Narcissistic Personality Inventory; EQ VAS = The EuroQuol Visual Analogue Scale; SWLS = Satisfaction with Life Scale; F-SozU = Questionnaire Social Support; DASS = Depression Anxiety Stress Scales; BFAS = Bergen Facebook Addiction Scale; SNS = social networking site; degrees of freedom of all F-values = 1,178; p = significance.

**Table 2:** Descriptive statistics (frequencies) of media use (T1 and T2).

		% at T1	% at T2
**Internet use**	(0) “never”	0	0
	(1) “less than once a month”	0	0.6
	(2) “once or twice a month”	0.6	0
	(3) “once a week”	2.8	1.7
	(4) “once or twice a week”	3.9	3.9
	(5) “once a day”	16.8	14.5
	(6) “more than once a day”	76.0	79.3
**SNSs use**	(0) “never”	0	0
	(1) “less than once a month”	5.0	3.9
	(2) “once or twice a month”	2.8	5.6
	(3) “once a week”	10.1	10.6
	(4) “once or twice a week”	19.6	16.8
	(5) “once a day”	23.5	33.0
	(6) “more than once a day”	39.1	30.2
**Used SNSs**	Facebook	100.0	100.0
	Twitter	8.9	10.1
	Instagram	24.0	31.3
	Tumblr	7.3	5.6
	Other	7.8	11.2

N = 179; SNS = social networking site; due to rounding, the sum of listed figures may be higher/lower than 100%.

Due to the polythetic scoring, eight (4.5%) participants reached the critical cutoff score at T1 and 15 (8.4%) participants reached it at T2. According to the monothetic scoring, the critical cutoff score occurred for one (0.6%) participant at T1 and for three (1.7%) participants at T2. Considering the specific addiction content of the six FAD items, their descriptive values were analyzed separately (see Table 3). The response range of all items at T1 was 1 to 4, the range of all items at T2 was 1 to 5. The mean values did not differ significantly. However, it is conspicuous that while at T1 a value ≥ 3 for Item 5 (withdrawal) was reached by 2.2% of the participants (value 3: three persons; value 4: one person), at T2 7.3% of the participants reached a value ≥ 3 for this item (value 3: nine persons; value 4: three persons; value 5: one person).

**Table 3:** Descriptive statistics and mean comparison between T1 and T2 of the BFAS items (within-subjects ANOVA).

	T1		T2			
	M (SD)	≥ 3: N (%)	M (SD)	≥ 3: N (%)	F	p
Item 1 (salience)	2.01 (.98)	58 (32.6)	2.08 (1.07)	58 (32.6)	.837	.362
Item 2 (tolerance)	1.80 (.89)	40 (22.3)	1.77 (.90)	37 (20.7)	.219	.641
Item 3 (mood modification)	1.51 (.84)	27 (15.1)	1.65 (.94)	33 (18.4)	3.387	.067
Item 4 (relapse)	1.63 (.91)	34 (19.0)	1.59 (.90)	31 (17.3)	.380	.539
Item 5 (withdrawal)	1.23 (.50)	4 (2.2)	1.26 (.68)	13 (7.3)	.359	.550
Item 6 (conflict)	1.35 (.67)	16 (8.9)	1.42 (.80)	17 (9.5)	.899	.344

N = 179; M = Mean; SD = Standard Deviation; degrees of freedom of all F-values = 1,178; p = significance; response range of all items: T1: 1–4, T2: 1–5.

### Associations of FAD with media use, personality, mental and physical health variables

At T1, FAD correlated significantly positively with SNSs use (r = .42, p < .001). The correlations with the other investigated variables did not become significant. In contrast, at T2, FAD was significantly positively related to SNSs use (r = .37, p < .001), narcissism (r = .26, p < .001), depression (r = .22, p < .01), anxiety (r = .32, p < .001), and stress symptoms (r = .20, p < .01). When comparing these correlations between T1 and T2, the correlation between FAD and anxiety symptoms (at T1: r = .02, n.s.) showed the highest significant change (effect size: Cohen’s q = .32, medium effect; see [57]). At T2, there was also a significant positive correlation between narcissism and stress symptoms (r = .16, p < .05). A cross-time calculation which included FAD at T2 and all other investigated variables at T1 showed that FAD was significantly positively correlated with SNSs use (r = .33, p < .001) and with narcissism (r = .19, p < .05). FAD at T1 was significantly positively related to SNSs use at T2 (r = .33, p < .001).

Based on the significant positive correlations between depression and anxiety symptoms, and FAD at T2, and earlier studies which described depression and anxiety symptoms to be possible predictors of FAD [11, 18, 58], a multiple linear regression analysis was calculated. Following earlier research (e.g., [11]), the regression model included depression and anxiety symptoms as independent variables and FAD as the dependent variable, controlling for the variables gender and age. There was no violation of multicollinearity assumption: all values of tolerance were > .25, and all variance inflation factor values were < 5 (see [59]). The model explained 10.7% of the variance, F(4,174) = 5.230, p < .01. Only anxiety symptoms showed a significant result (standardized beta = .310, p < .01; 95% CI [.142;.587]).

In the next step, the relationship between narcissism and FAD at T2 was investigated in more detail. Narcissism correlated significantly positively with most of the FAD items (Item 1, salience: r = .23, p < .01; Item 2, tolerance: r = .18, p < .05; Item 4, relapse: r = .20, p < .01; Item 5, withdrawal: r = .27, p < .001; Item 6, conflict: r = .16, p < .05). Only the relationship with Item 3 (mood modification) did not become significant (r = .11, n.s.).

A regression model that included narcissism as the independent variable and FAD as the dependent variable, controlling for the variables gender and age, explained 7.1% of the variance, F(3,175) = 4.450, p < .01. While gender and age showed no significant results, the result for narcissism became significant (standardized beta = .259, p < .001; 95% CI [.187;.655]).

### Mediation analysis

**Fig 2:**
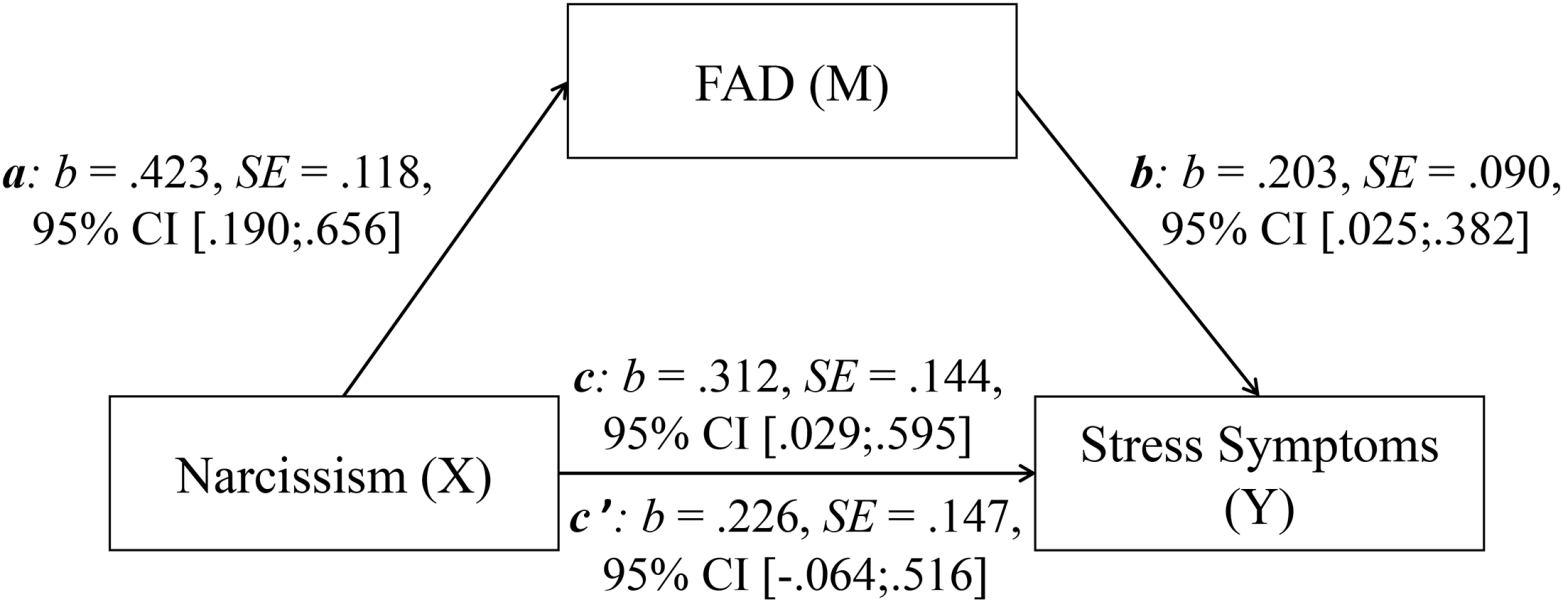
Mediation model including results. *c* = total effect, *c’* = direct effect; *b* = standardized regression coefficient, SE = standard error, CI = confidence interval.

As presented in Fig 2, the bootstrapped mediation analysis shows that FAD fully mediates the relationship between narcissism and stress symptoms. While path *c* (total effect) is significant (p < .001), path *c’* (direct effect) which implies the inclusion of FAD in the model does not become significant (p = .125). The indirect effect (*ab*) becomes significant, *b* = .086, SE = .046, 95% CI [.018;.204]; P_M_: *b* = .275, SE = 6.614, 95% CI [.024;2.509].

## Discussion

The present study belongs to the first longitudinal works to investigate FAD and its relationships with personality, mental health and physical health in Germany. Considering that only little is known about the development and maintenance of FAD, the present work included two time points of measurement of all investigated variables in order to assess the course of FAD and its associations. We found significant results that contribute to the better understanding of FAD.

Mean FAD values (T1 and T2) for our German student sample were remarkably lower than the value reported by Andreassen et al. [20] (M = 13.00, SD = 5.20) in a student sample in Norway, where Facebook in terms of percentage has almost twice as many users as in Germany (www.internetworldstats.com/stats4.htm).

Even though we did not find significant change in the mean FAD level after one year, the number of participants who reached a critical FAD score increased remarkably (polythetic scoring: 4.5% to 8.4%; monothetic scoring: 0.6% to 1.7%). Particularly, it is important to note that remarkably more participants had higher values of the withdrawal item at T2 than at T1. This emphasizes the enhanced meaning of psychological withdrawal in problematic Facebook use: More and more users become nervous without the possibility to use Facebook (see also [19]). This fits earlier research which described psychological withdrawal after cessation of contact with Internet as one of the main symptoms of problematic Internet use [60]. Increased withdrawal could be positively related to the so-called “Fear of Missing out (FoMo)”: the fear to miss important social information and to lose popularity, often described by Facebook users who are unable to use the SNS as often as desired. FoMo has been found to positively mediate the relationship of the motive need to belong and the motive need for popularity with Facebook use. Furthermore, it was positively associated with perceived stress symptoms related to Facebook use [61, 62].

While our hypotheses were partly confirmed at T2, at T1, FAD was not significantly related to the investigated variables. This could be partly because significantly more participants reached the critical cutoff score at T2 than at T1. Thus, at T1, FAD had a weaker association with the participants’ life and mental health than at T2. Furthermore, before drawing final conclusions, these differences underline the necessity of longitudinal observations of the course of FAD and of its associations that seem to change over time.

Our results indicate that people who intensively use SNSs can be at risk to develop FAD. However, general Internet use was not significantly associated with FAD underlining the need to differentiate between the types of online activities when investigating media use. According to earlier research, at T2 FAD, was positively associated with the three negative mental health variables (confirming Hypothesis 1). The comparison between the correlations at T1 and at T2 indicated that especially the positive association between FAD and anxiety symptoms increased over time. The role of anxiety symptoms in terms of FAD, also described by earlier studies (e.g., [11]), was underlined by the results of the regression analysis. Interestingly, of all FAD items, the withdrawal item showed the highest significant positive correlation with anxiety symptoms (r = .34, p < .001). Thus, it could be assumed that people with increased anxiety symptoms, who often use Facebook to find relief and to escape (see [4]), have an enhanced probability to develop FAD. Because of their anxiety symptoms, they are often nervous and worried about the consequences of their behavior. Therefore, withdrawal is one of their main symptoms, especially because they are afraid to miss out on things when not using Facebook. However, we did not measure FoMo or any other specific Facebook related form of anxiety. So, this possible interpretation of our results remains open for discussion.

While FAD was positively related to the negative mental health variables at T2, none of the positive mental health variables was significantly associated with FAD (contradicting Hypothesis 2). Such differing results speak for the dual-factor model of mental health which emphases positive and negative mental health to be interrelated but separate unipolar dimensions of general mental health [63, 64]. Furthermore, even though we found a significant decrease in physical health after one year, FAD does not seem to be directly related to physical health (contradicting Hypothesis 3).

Our results could partly be due to the fact that even though the critical cutoff score at T2 was reached by a significantly higher number of participants than at T1, most of our participants had mean FAD values under the critical cutoff. Therefore, most of them do not suffer directly from the consequences of FAD, on the one hand, and experience, on the other hand, the benefits of Facebook use. For example, some studies reported a positive association between social support and Facebook use, especially the number of Facebook-friends [34, 65]. However, as the few conducted longitudinal studies show, persistent Facebook use can negatively influence life satisfaction and physical health (e.g., [28]).

In accordance with our expectations, we found a positive relationship between narcissism and FAD (confirming Hypothesis 4). Moreover, FAD fully mediated the association between narcissism and stress symptoms (confirming Hypothesis 5). Therefore, FAD could be a potential risk factor for people with elevated values of narcissism. Facebook use holds a particular meaning for narcissistic people. On Facebook, they can quickly initiate many superficial relationships with new Facebook-friends and get a large audience for their well-planned self-presentation. The more Facebook-friends they have, the higher is the possibility that they attain the popularity and admiration they are seeking for; whereas in the offline world they might not be as popular since their interaction partners can quickly perceive their low agreeableness and exaggerated sense of self-importance [32, 33, 66]. Narcissistic people use the positive feedback from interaction partners to regulate their self-esteem and for self-enhancement [67]. Therefore, it can be assumed that narcissistic users spend more time thinking about Facebook than others—planning their online self-presentation and interaction and reflecting received feedback. Thus, although Facebook use is very attractive for narcissists, it could make them especially vulnerable to FAD. Correspondingly, at T2, narcissism correlated significantly positively with most of the FAD items. The highest positive associations were found for the items withdrawal, salience, and relapse.

Moreover, our results indicate that FAD mediates the relationship between narcissism and stress symptoms. One possible interpretation is that narcissists plan their self-presentation to impress their audience. The larger the audience, the more difficult it is to impress all interaction partners, and the probability to receive negative feedback increases. This increases the self-presentation efforts of narcissistic users and the time they spend thinking about and using Facebook, which in turn increases their vulnerability to FAD. As their FAD level increases, they experience more symptoms like withdrawal and relapse, which enhance their stress symptoms. This interpretation is open for discussion and should be considered with caution, especially in light of the low internal consistency of the used narcissism scale and the brief measure of FAD with only six items.

### Limitations and further research

Surely our study has some limitations which reduce the generalizability of our results and the conclusions that can be drawn from them. We worked with a student sample including mostly female Facebook users. In order to at least partially tackle this limitation, we compared the presented results of the zero-order bivariate correlations between FAD and the other investigated variables at T1 and at T2 with the results of appropriate partial correlations controlling for gender. No significant differences between the two kinds of correlations were found (all comparisons: q < .10, [57]). Nevertheless, the composition of our sample limits the generalizability of the present results. Therefore, future studies should investigate their replicability using a larger and more representative sample with an equal gender ratio.

Present data were collected by online self-report measures that, despite of the guarantee of anonymity, are prone to social desirability. Therefore, we advise future studies with a similar design to include an instrument measuring the tendency of social desirability, for example the Balanced Inventory of Desirable Responding (BIDR) [68], to control the effect of social desirability post hoc in the calculations.

As already mentioned, to measure FAD, we used the short version of the Bergen Facebook Addiction Scale, a self-report measure with only six items. This scale has been reported to have similarly good psychometric properties as the long version [15, 20, 48]. In the present study, it showed satisfactory to good reliability values. Nevertheless, to meet the multifaceted nature of FAD and to improve validity of the measurement, we advise further investigations to focus on the development of more complex instruments to measure FAD. Considering that especially addicted people tend to underestimate the level of their addictive behavior, objective measures and observations should be included to assess FAD. Furthermore, taking into account that physiological functions such as blood pressure and heart rate have been demonstrated to be associated with problematic Internet use [5], a focus should also be laid on potential physiological markers of FAD.

Interestingly, the FAD item mood modification was not significantly related to narcissism, although narcissistic individuals receive increased attention and positive feedback on Facebook which might increase their positive mood [69], and, furthermore, could enhance their Facebook use frequency and risk to develop FAD. One reason for this might be that narcissistic people experience a short-term mood modification by Facebook use which is not measurable by the single FAD item. To investigate the relationship between mood modification, narcissism and FAD more specifically, further measures like the Positive and Negative Affect Schedule (PANAS) [70]–often used in studies demonstrating significant associations between problematic Internet use and mood (e.g., [5, 9])–should be included to assess mood before and after Facebook use.

The present study is the first step in the investigation of FAD in Germany. Considering the results of studies showing that various activities on Facebook can differently influence mental health [71, 72], future work should focus on the duration and frequency of Facebook use and individual Facebook activities. This would further contribute to the understanding of the development and the maintenance of FAD. Furthermore, considering that Facebook is the most popular, but often not the only, used SNS (see Table 2), the frequency of the use of other SNSs should be included in future investigations.

To sum up, the present results give the first overview of FAD in Germany, underlining the huge need for further investigations in this research field. Our one-year follow-up shows that significantly more people reach the critical cutoff score compared to the previous year, and that negative mental health values, especially anxiety symptoms, are positively associated with FAD. However, to draw generalizable conclusions, the present results should be replicated in a larger, age and gender representative sample using additional measures beyond self-report scales.

## Supporting information

S1 Dataset Dataset used for analyses in present study. (SAV)
